# Asthma Pregnancy Alters Postnatal Development of Chromaffin Cells in the Rat Adrenal Medulla

**DOI:** 10.1371/journal.pone.0020337

**Published:** 2011-05-27

**Authors:** Xiu-Ming Wu, Cheng-Ping Hu, Xiao-Zhao Li, Ye-Qiang Zou, Jun-Tao Zou, Yuan-Yuan Li, Jun-Tao Feng

**Affiliations:** Department of Respiratory Medicine, Xiangya Hospital, Central South University, Changsha, Hunan, China; University of Giessen Lung Center, Germany

## Abstract

**Background:**

Adrenal neuroendocrine plays an important role in asthma. The activity of the sympathoadrenal system could be altered by early life events. The effects of maternal asthma during pregnancy on the adrenal medulla of offspring remain unknown.

**Methodology/Principal Findings:**

This study aims to explore the influence of maternal asthma during pregnancy on the development and function of adrenal medulla in offspring from postnatal day 3 (P3) to postnatal day 60 (P60). Asthmatic pregnant rats (AP), nerve growth factor (NGF)-treated pregnant rats (NP) and NGF antibody-treated pregnant rats (ANP) were sensitized and challenged with ovalbumin (OVA); NP and ANP were treated with NGF and NGF antibody respectively. Offspring rats from the maternal group were divided into four groups: offspring from control pregnant rats (OCP), offspring from AP (OAP), offspring from NP (ONP), and offspring from ANP (OANP). The expressions of phenylethanolamine N-methyltransferase (PNMT) protein in adrenal medulla were analyzed. The concentrations of epinephrine (EPI), corticosterone and NGF in serum were measured. Adrenal medulla chromaffin cells (AMCC) were prone to differentiate into sympathetic nerve cells in OAP and ONP. Both EPI and PNMT were decreased in OAP from P3 to P14, and then reached normal level gradually from P30 to P60, which were lower from birth to adulthood in ONP. Corticosterone concentration increased significantly in OAP and ONP.

**Conclusion/Significance:**

Asthma pregnancy may promote AMCC to differentiate into sympathetic neurons in offspring rats and inhibit the synthesis of EPI, resulting in dysfunction of bronchial relaxation.

## Introduction

Asthma is a disease with its origins in early life. Maternal asthma is a risk factor for asthma in children [1]. Study demonstrated that some components in uterus or early postnatal environment might cause increase of asthma susceptibility in offspring [2]. Epigenetic studies suggested that environmental factors exposed to pregnant mothers were closely related to the childhood asthmatic phenotypes, especially after the birth [3], [4]. However, the pathogenesis is complex and not entirely understood.

Adrenal neuroendocrine played an important role in the regulation of bronchial diastole by secreting epinephrine (EPI) [5]. Recent reports demonstrated that the activity of the sympathoadrenal system could be altered by early life event; Sympathetic adrenal cells, derived from embryonic neural crest stem cells, could migrate and locate into adrenal gland during the development [6]. After the lost of neurons features, those cells differentiate into adrenal medulla chromaffin cells (AMCC) with endocrine function [7]. However, experimental study indicated that AMCC have redundant functions in the setting of abnormal physiological functions, including loss of endocrine phenotype and acquisition of neuronal properties [8], [9]. Studies indicated that continuous infusion of nerve growth factor (NGF) into 17 days pregnant rats enhanced the transformation of AMCC into sympathetic neurons, which then infiltrated into adrenal cortex and medulla and altered their structures [10].The alteration in the early sympathetic-adrenal system activity, development, and maturation partly attribute to environmental stimulation during uterus and after birth[11], [12]. The development and maintenance of AMCC are critically dependent NGF. Circulating NGF levels are increased in humans with allergic diseases and asthma [13]. Investigation recently found that increased NGF in asthma could induce functional redundancy of rat AMCC, which resulting in transforming them into sympathetic neurons, and significantly reduced the synthesis and release of EPI, unbalancing bronchial contraction and relaxation[14], [15].Exposure to high level of NGF in the intrauterine environment may play an important role in the process of neural stem cell growth, migration and development and the differentiation of AMCC into sympathetic neurons, interfering with the synthesis, storage, release of EPI, even participating in adult bronchial asthma. So far, the influence of intrauterine environment during asthma attack on the development and redundant function of the adrenal medulla which adjust bronchial diastole by EPI has not been reported. We presume that the incidence of asthma during pregnancy alter the differentiation of AMCC and initiate the redundant functions of AMCC in offspring rats.

To test this hypothesis, we observed the structure and function of adrenal medulla in offspring rat in different period (birth, early youth, adolescence, adulthood), to detect the effect of maternal asthma during pregnancy on the development of adrenal medulla in their offspring.

## Results

### Airway responsiveness to histamine

**Figure 1:**
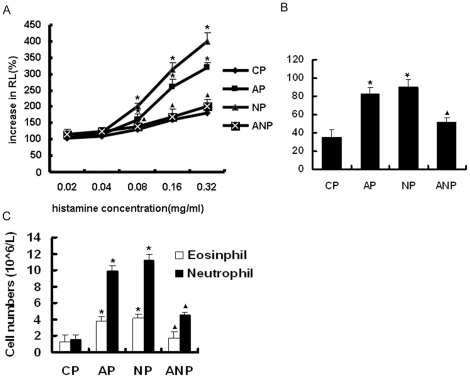
The changes of airway resistance (RL) and the cell counts in the BALF of maternal rats. **A**: RL in maternal rats. **B**: The total cell counts in the BALF of maternal rats. C: The number of neutrophils and eosinophils in BALF of maternal rats. The values are means ± SEM (n = 8); * *P*<0.05 vs. CP, ^▴^<0.05 vs. AP.

With the increasing concentration of histamine, airway resistance (RL) was gradually increased in each group. The RL was significantly increased in AP and NP group compared with CP group when the concentration of histamine reached 0.08 mg/ml and above (*P*<0.05 ).ANP group were decreased in RL compared with AP group (*P*<0.05 )(Figure 1A).

### Total and differential white cell counts of BALF

Total cell counts were significantly increased in AP and NP group compared with the CP group (P<0.05). There is significant decrease in ANP rats compared to AP group (P<0.05) (Figure 1B) The AP and NP group also have significantly greater numbers of neutrophils and eosinophils in the BALF than the CP group (*P*<0.05 ). There is significant decrease in ANP rats compared to AP group(P<0.05) (Figure 1C).

### Lung tissue morphology of maternal rats

**Figure 2:**
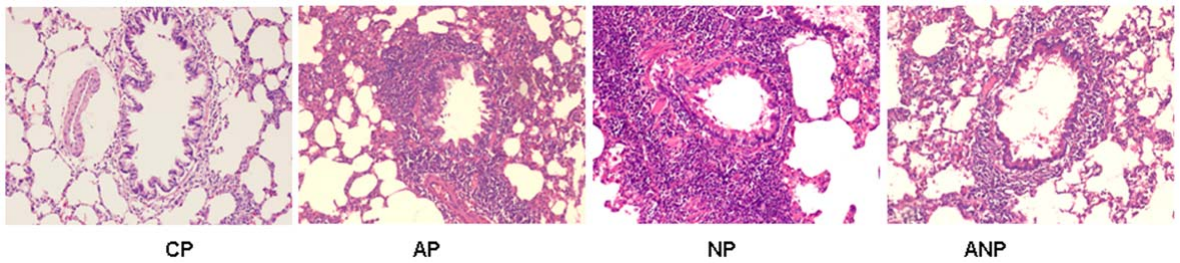
Histopathology examination of lung in each maternal group. No obvious lesions of airway structure were appeared in CP rats. Bronchial epithelial shedding, eosinophil and neutrophils infiltration surrounding airway were found in AP rats, and these pathological changes were aggravated in NP rats and alleviated in ANP rats. (The magnification of the image is 100 ×).

Under microscope, the airway structure was undamaged in CP rats, and the infiltration of inflammatory cell was not appeared around bronchia and vessel. While bronchial epithelial shedding, increased mucus plug, eosinophils and neutrophils infiltration surrounding airway were found in AP rats. The inflammatory cell infiltration is more obvious in bronchial wall in NP group compared with AP group, while such pathological changes in lung tissue significantly relieved in ANP group. (Figure 2)

### Adrenal medulla alteration in maternal rats

**Figure 3:**
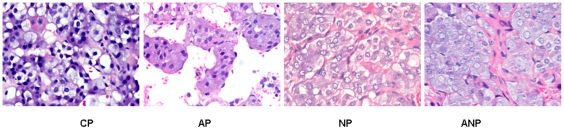
Histopathology examination of adrenal medulla in maternal rats. The shape of adrenal medullar cell was regular, and damaged structure was not observed in CP rats. Adrenal medulla vacuolar degeneration and lipid increases were observed in AP rats, which was aggravated in NP rats and alleviated in ANP rats. (The magnification of the image is 400 ×).

Under microscope, the shape of adrenal medullar cell was regular, and no obvious pathological changes were found in CP rats. The increase of vacuolar degeneration and lipid were observed in AP rats adrenal medulla cells, NGF intervention aggravated while NGF antibody intervention alleviated those lesions (Figure 3).

**Figure 4:**
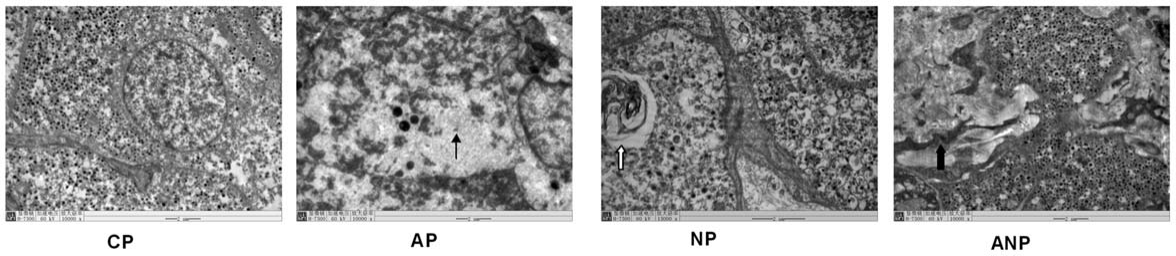
Electron micrograph of adrenal medulla in maternal rats. AMCC lined up tightly in order, containing round nucleus, abundant chromatin granules, and mitochondrion with clear structures in CP rats. Mitochondrion swelled, lipid increased and chromaffin granules decreased in AP and NP rats (thin arrow). Cytoplasm lamellar-like structure appeared in NP rats (hollow arrow); Lesions were alleviated and appeared more collagen tissue, which divided adrenal medulla into island in AP rats (thick arrow). (The magnification of the image is 10000 ×).

Electron microscopy indicated that AMCC lined up tightly in order,contained round nucleus, abundant chromaffin granules, mitochondrion with clear structures in CP rats. AMCC presented signs of lesions: swelling mitochondrion, increased lipid, decreased chromaffin granules in AP and NP rats. Interestingly, cytoplasm lamellar-like structure was found in NP rats; NGF antibody treatment improved such pathological changes, promoted the deposition of collagen tissue, which divided adrenal medulla cells into island (Figure 4).

### Adrenal medulla changes in offspring rats

**Figure 5:**
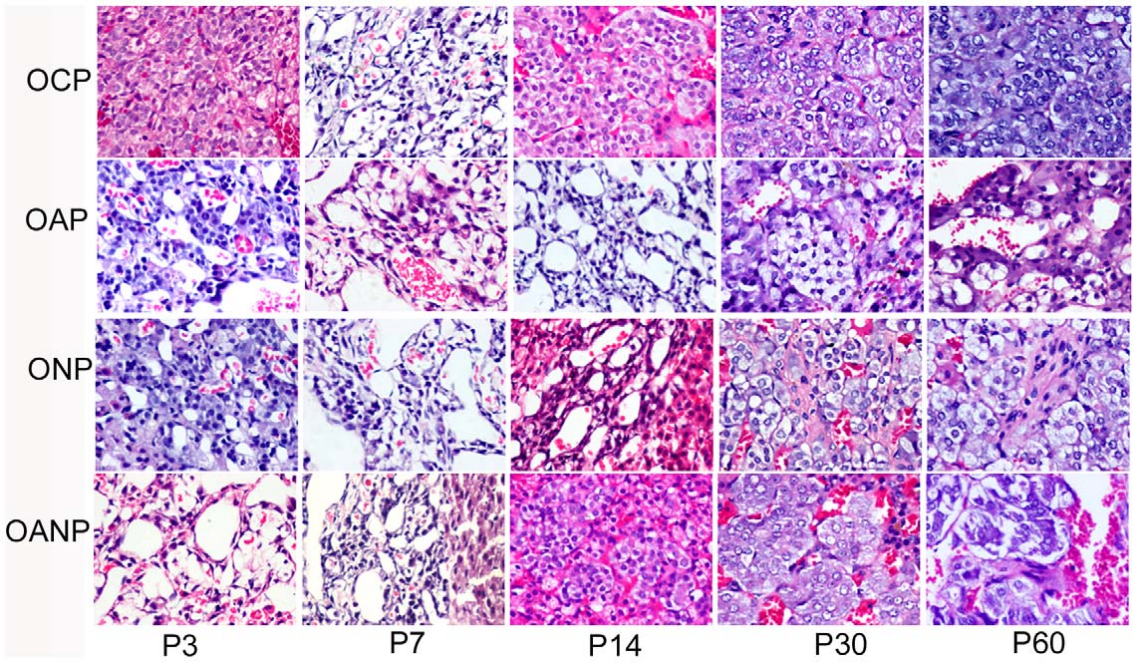
Histopathology examination of adrenal medulla in offspring rat. P3:postnatal day 3;P7:postnatal day 7; P14:postnatal day 14; P30:postnatal day 30; P60:postnatal day 60; Adrenal medulla cells showed cytoplasm edema, some spindle shape chromaffin cells with decreased particles and a small amount of connective tissue in OAP and ONP rats from P3 to P60. AMCC decreased and a large number of connective tissues separated medulla from P3 to P60 in OANP rats. (The magnification of the image is 400 ×).

**Figure 6:**
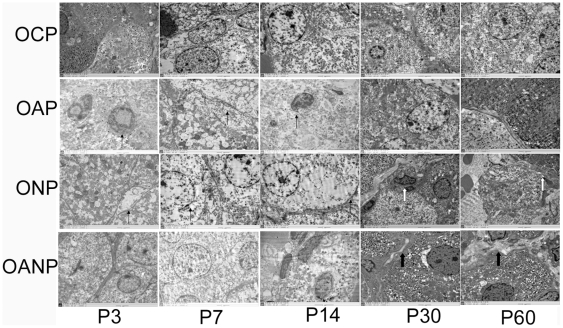
Electron micrograph of adrenal medulla in offspring rat. The adrenal medulla cells of OAP and ONP rats showed edema of cytoplasm and mitochondrial, vacuolar degeneration, deceased EPI secretory granule. chromaffin cells appeared fiber outgrowth and changed into spindle shape with long fusiform nucleus from P3 to P14(thin arrow). From P30 to P60, vacuolar degeneration showed decreased and the PEI secretory granule become increasing. At the same time, nerve fibers of ONP rats were rich in density, myelinated and unmyelinated nerve fibers can be seen (hollow arrow). There were mitochondrial edema and decreased chromaffin with enriched EPI granules in OANP rats. More collagen emerged and divided adrenal medulla into island from P30 to P60 (thick arrow). (The magnification of the image is 10000 ×).

On P3: adrenal medulla cells scattered in zona reticularis and cytoplasm increased gradually in OCP rats, and electron micrograph revealed clear chromaffin granules (mainly adrenaline cells), rich lysosomes, mitochondria and sympathetic ganglion. OAP and ONP rats showed vacuolar degeneration, mitochondrial edema, and fiber outgrowth, decreased or lost of EPI chromaffin granules in adrenal medulla cells. In OANP rats, the numbers of medulla chromaffin cells were significantly decreased, but their organelles were still abundant (Figure 5, Figure 6).

From P7 to P14:The development of adrenal medulla in OCP rats has become matured, showing a small amount of connective tissue, blood vessels and a few sympathetic ganglion cells and medulla chromaffin cells, which were arranged in groups or cords. The adrenal medulla cells of OAP and ONP rats still indicated edema of cytoplasm and mitochondrion, vacuolar degeneration and decreased or lost of EPI secretory granule with lightly stained color. Some spindle shape chromaffin cells with long fusiform nucleus as well as a small amount of connective tissue were found in OAP and ONP rats (Figure 5, Figure 6).

From P30 to P60: OAP and ONP rats showed EPI secretory granule in medulla chromaffin cells become increasing. There also exist small amounts of connective tissue. ONP rats demonstrated more sympathetic ganglion cells and nerve fibers in adrenal medulla, where myelinated and unmyelinated nerve fibers could be observed. Adrenal medulla also appeared mitochondrial edema and decreased chromaffin cells with enriched EPI granules in OANP rats while more collagen emerged and divided adrenal medulla into island (Figure 5, Figure 6).

### The levels of serum EPI in maternal rats and their offspring

**Figure 7:**
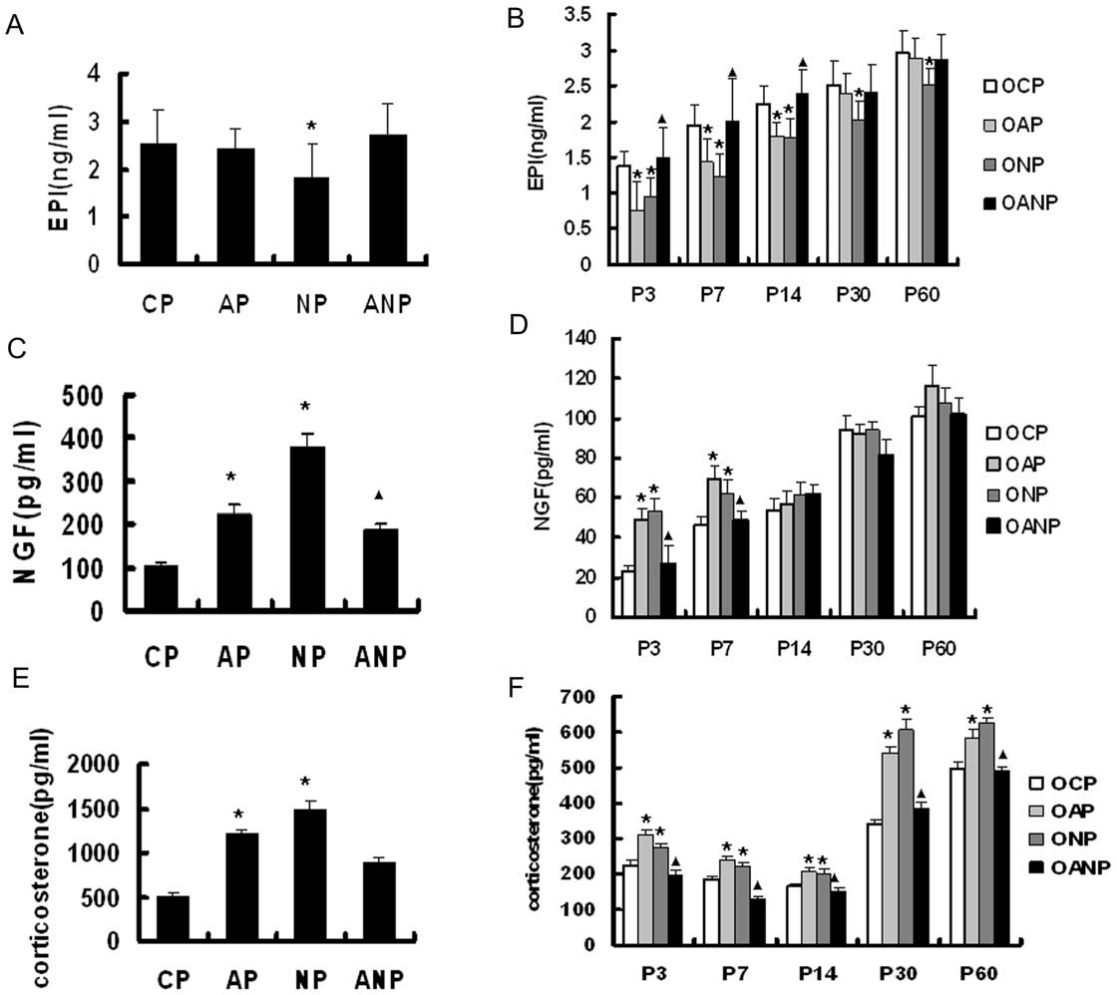
Serum levels of EPI, NGF and corticosterone in rats. **A**: Serum levels of EPI decreased significantly in NP rats compared to CP rats. **B**: Serum levels of EPI decreased in OAP rats from P3 to P14 compared to OCP rats and regained normal level from P30 to P60; however, in ONP rats, from P3 to P60, serum levels of EPI were lower than those in OCP rats; there is significant increase in OANP rats compared to OAP rats from P3 to P14. **C**: Serum levels of NGF were significantly increased in AP and NP rats compared to CP rats. Serum NGF levels decreased in ANP rats compared with OAP rats. **D**: Compared to OCP rats, serum levels of NGF increased from P3 to P7 in OAP and ONP rats and regained normal level from P14 to P60. Compared with OAP rats, serum NGF levels in OANP rats were lower from P3 to P7 and no significant difference from P14 to P60. **E**: Serum levels corticosterone increased in AP and NP rats compared to CP rats, while ANP rats showed a decline tendency compared to AP rats. **F**: Serum levels of corticosterone in OAP and ONP rats increased significantly compared to OCP rats from P3 to P60, while those in OANP rats were lower compared to OAP. Values are expressed as mean ±SEM(n = 8); * P<0.05 vs CP/OCP, ^▴^P<0.05 vs AP/OAP.

Compared with CP group, serum EPI levels in AP rats were not significantly increased, while serum EPI level in NP rats was further deceased compared to CP rats(P<0.05). After anti-NGF treatment, serum levels of EPI increased slightly in ANP rats (Figure 7 A).

Serum EPI levels were dramatically decreased in OAP and ONP rats from P3 to P14 compared to OCP rats(P<0.05), but then increased gradually from P30 to P60 in OAP rats, reaching value approaching those of OCP rats. However, serum EPI levels were lower in ONP rats at all developmental stages. There is significant increase in OANP rats compared to OAP rats from P3 to P14 (P<0.05) (Figure 7B).

### The levels of serum NGF in maternal rats and their offspring

Serum NGF levels were significantly increased in AP and NP rats compared with CP rats(P<0.05). Serum NGF levels decreased slightly in ANP rats after injecting anti-NGF, but it didn't recover to normal level (Figure 7C).

Serum NGF levels were increased from P3 to P7 in OAP and ONP rats compared with OCP rats (<0.05).NGF levels became restoration from P14 to P60. Compared with OAP rats, serum NGF levels in OANP rats were lower from P3 to P7(P<0.05) and no significant difference from P14 to P60. (Figure 7D).

### The levels of serum corticosterone in maternal rats and their offspring

Results showed that serum corticosterone levels increased in AP and NP rats compared with CP rats (P<0.05), while serum corticosterone levels decreased slightly in ANP rats (Figure 7E).

From P3 to P60, serum corticosterone levels in OAP and ONP rats increased significantly compared to OCP rats (P<0.05), while those in OANP rats were lower compared to OAP rats (P<0.05) (Figure 7F).

### The expression of PNMT in adrenal medulla

**Figure 8:**
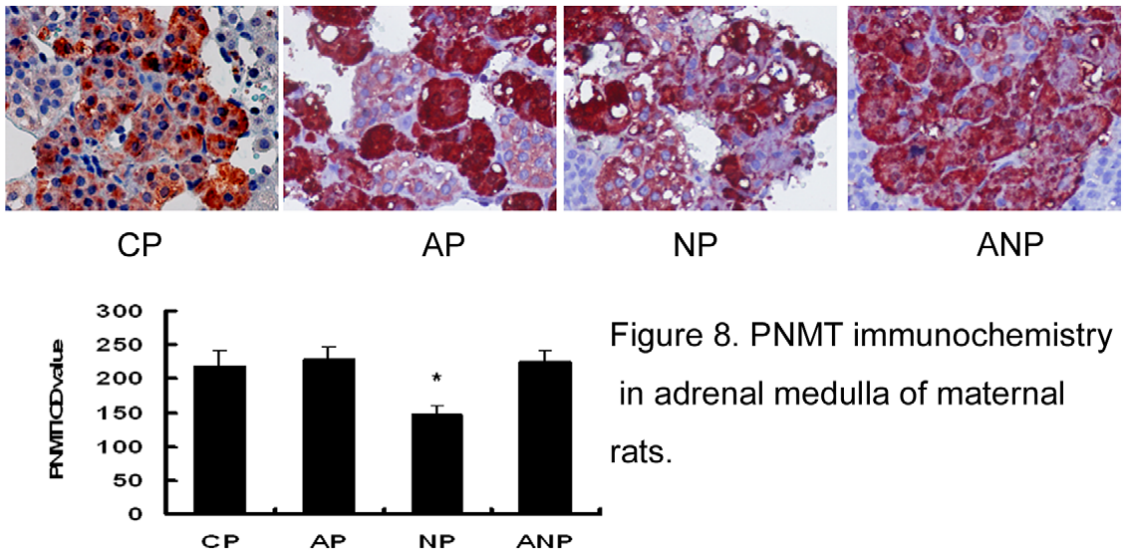
PNMT immunochemistry in adrenal medulla of maternal rats. The expression of PNMT decreased in NP rats significantly compared with CP rats. IOD values are expressed as mean ±SEM(n = 8); * P<0.05 vs CP. (The magnification of the image is 400 ×).

**Figure 9:**
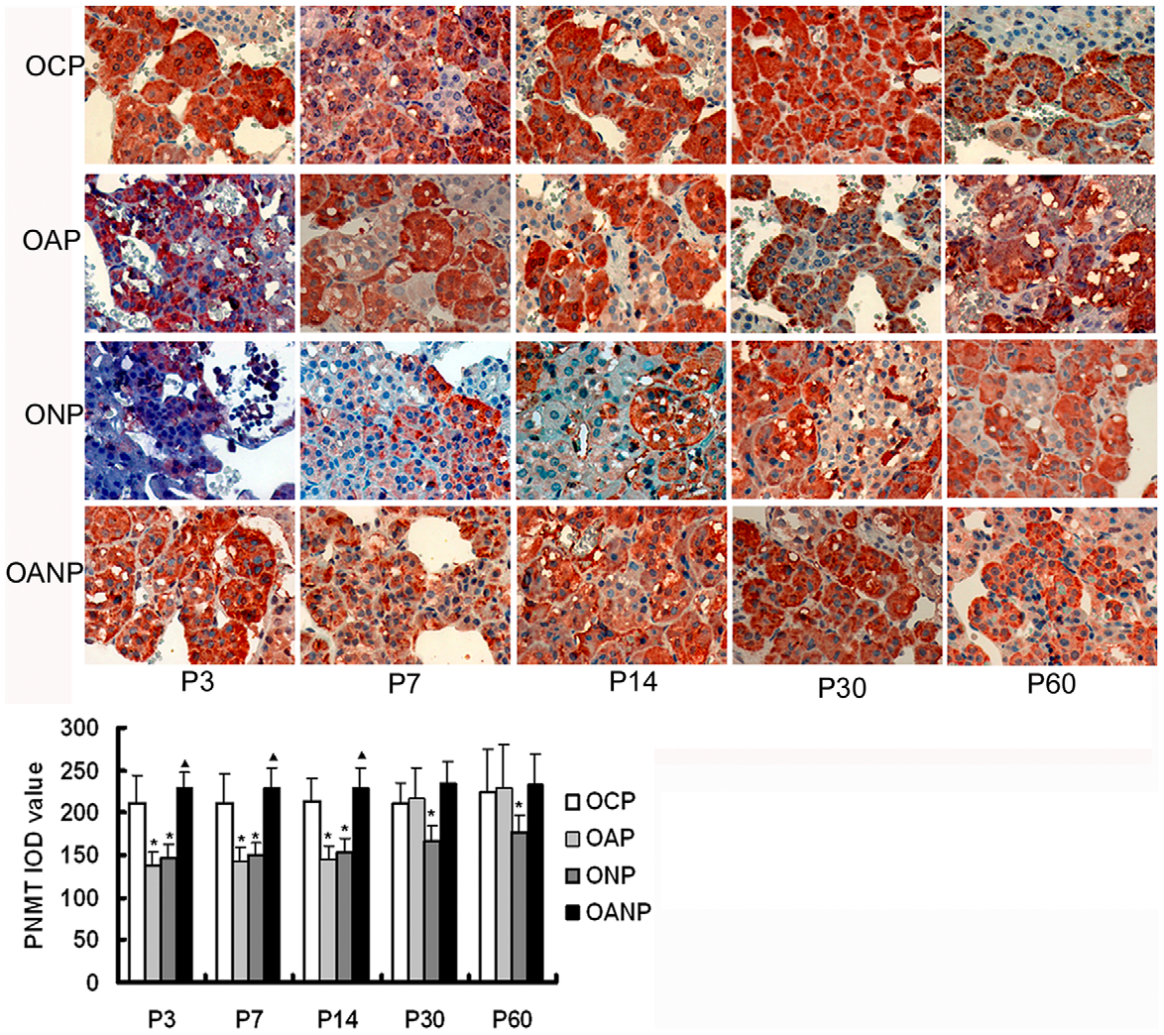
PNMT immunochemistry in adrenal medulla of offspring rats. The expression of PNMT protein in the OAP and ONP rats adrenal medulla decreased significantly compared to OCP rats from P3 to P14 and gradually increased in OAP rats from P 30 to P60, there was a trend towards a lower expression in ONP rats. The expressions of PNMT protein in OANP rats increased from P3 to P14 and showed no significant difference compared with OAP rats from P30 to P60. IOD values are expressed as mean ±SEM(n = 8); * P<0.05 vs. OCP, ^▴^<0.05 vs. OAP. (The magnification of the image is 400 ×).

In this study, immunohistochemistry results showed that the expressions of PNMT in NP rats decrease significantly compared with CP rats (P<0.05) (Figure 8). In the development of offspring, the expressions of PNMT protein in the OAP rats adrenal medulla decreased significantly when compared with OCP rats from P3 to P14 (P<0.05). The expressions of PNMT protein gradually increased gradually in OAP rats from P30 to P60; there was a trend towards a lower expression in ONP rats. However, the expressions of PNMT protein in OANP were increased from P3 to P14 (P<0.05) and showed no significant difference compared with OAP rats from P30 to P60 (Figure 9).

### The expression of NGF in adrenal medulla

**Figure 10:**
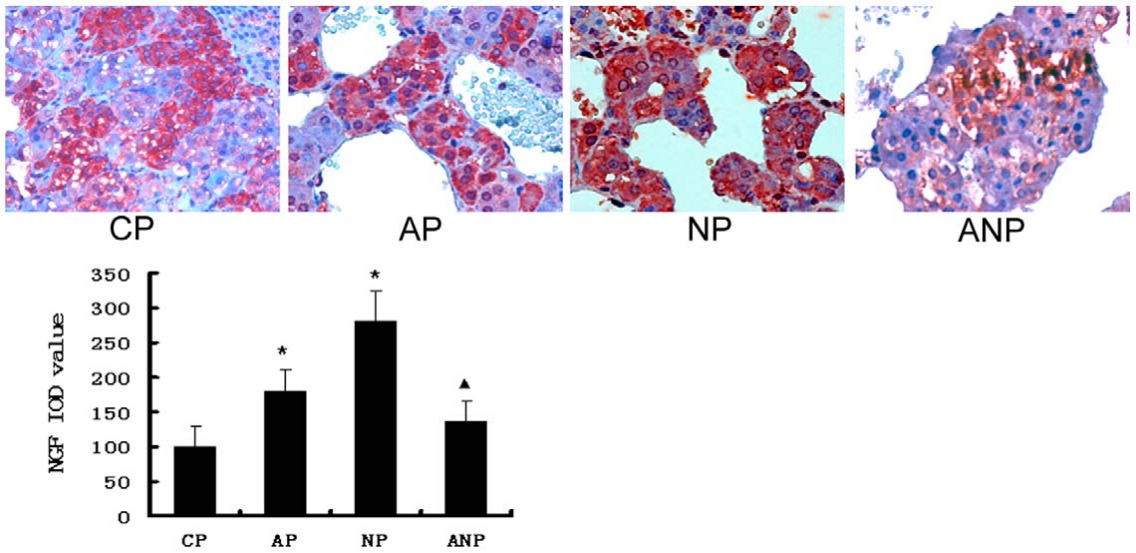
NGF immunochemistry in adrenal medulla of maternal rats. NGF protein in AP and NP rats were significantly higher than that in CP rats. NGF expression decreased in ANP rats compared with AP rats. IOD values are expressed as mean ±SEM(n = 8); * P<0.05 vs CP, ^▴^P<0.05 vs AP. (The magnification of the image is 400 ×).

The expressions of NGF protein were found both in cytoplasm and nucleus in adrenal medulla cells. Compared with the CP rats, NGF expression increased significantly in AP and NP rats (P<0.05). Serum NGF levels decreased in ANP rats compared with AP rats (P<0.05) (Figure 10).

**Figure 11:**
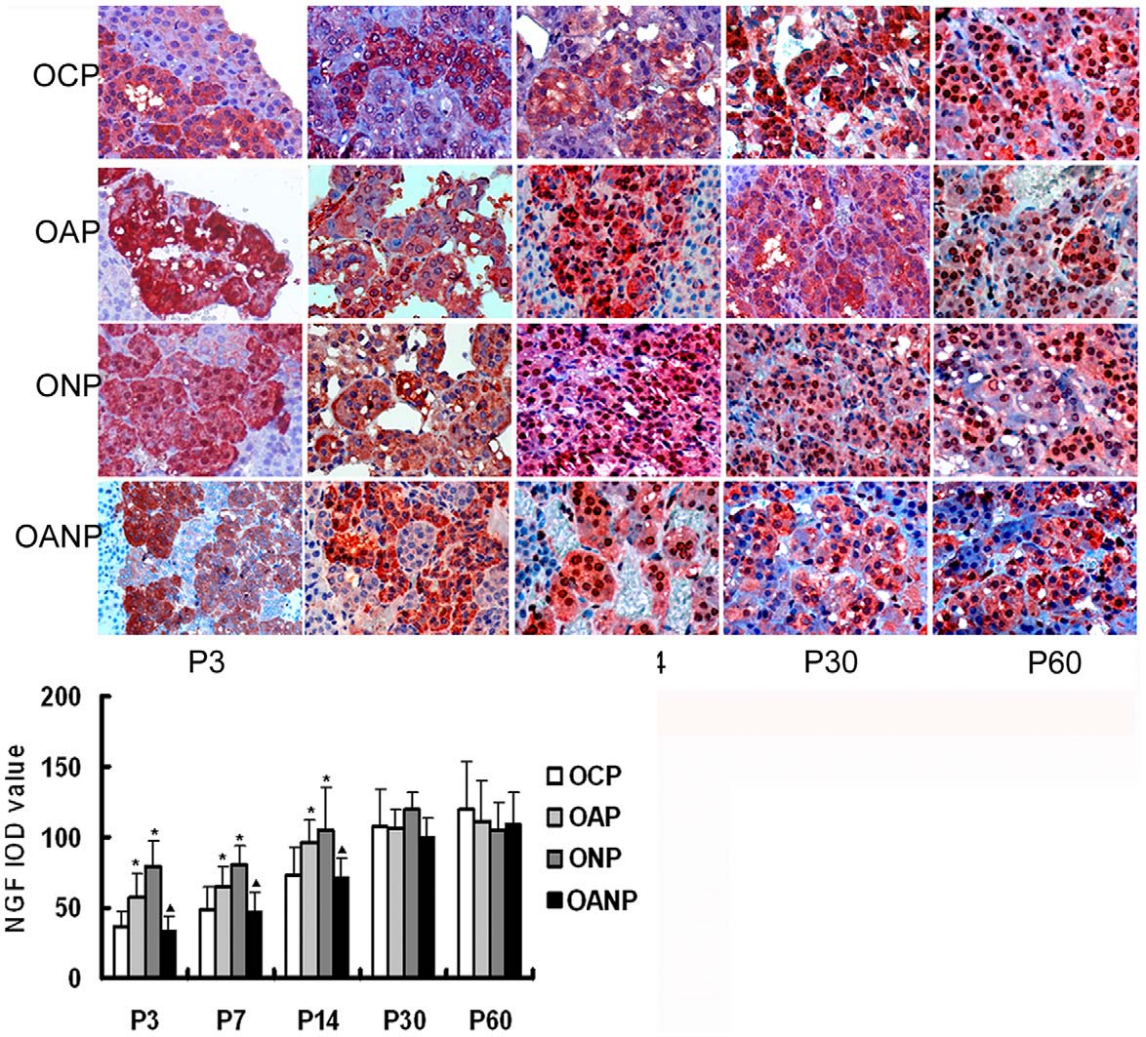
NGF immunochemistry in adrenal medulla of offspring rats. The expression of NGF increased in OAP and ONP rats adrenal medulla compared to OCP rats from P3 to P14 and return to normal level from P30 to P60. The expressions of NGF protein in OANP rats decreased from P3 to P14 compared to OAP. IOD values are expressed as mean ±SEM(n = 8); * P<0.05 vs. OCP, ^▴^<0.05 vs OAP. (The magnification of the image is 400 ×).

NGF immunoreactivity increased in OAP and ONP rats adrenal medulla compared to OCP rats from P3 to P14 (P<0.05)and remained normal level from P30 to P60. The expressions of NGF protein in OANP decreased from P3 to P14 compared to OAP rats (P<0.05) and no distinction between OANP rats and OAP rats from P30 to P60 (Figure 11).

## Discussion

The notion of fetal origins of adult disease hypothesis (FOAD) was presented through a series of epidemiological studies [16], [17]. The mechanisms may include early lesions in intrauterine that altered fetal organs permanently or procedurally during growth-sensitive period [18], [19], various hormone axis reset, which increased its susceptibility to various chronic diseases [20]. Recent report also proved that prenatal environmental exposures could induce respiratory disease associated systemic and airway immune changes in the adult offspring [21].

In our present studies, the structure and function of adrenal medulla in offspring rats from maternal asthma rats during pregnancy differed from normal maternal rats, including swelling AMCC, vacuolar degeneration, and the prone development of AMCC into sympathetic nerve cells:(1) increased cell size and spindle shape with long fusiform nucleus; (2) ultra structure of neurological type (fiber outgrowth) appeared, EPI secretary granules decreased and even disappeared, density and morphology changed; (3) lack of PNMT immunoreactivity and dysfunction of epinephrine synthesis and release; (4) adrenal medulla cells repaired in the form of progressive fibrosis with the growth of offspring. Environmental stimulation in uterus and after birth could alter the development and maturation of the early sympathetic-adrenal system [22]. It is well-known that AMCC possessing redundancy function could transform into the sympathetic adrenal nerve cells [23]. We speculated that the intrauterine environment with asthma attack could have an influence on the differentiation of AMCC into adrenal sympathetic nerve cells in offspring.

Studies demonstrated that NGF could be associated with asthma attack [24], [25]. Investigation recently found that increased NGF in asthma could induce functional redundancy of rat AMCC, which resulting in transforming them into sympathetic neurons, and significantly reduced the synthesis and release of EPI, unbalancing bronchial contraction and relaxation[14], [15].Our works found that high NGF level in asthma maternal serum and the morphology and function of AMCC in ONP rats were more worse maintained,which revealed that high concentrations of NGF exposure during pregnancy may initiate the transformation of AMCC into neurons in offspring rats. We concluded that the conversion of AMCC to neurons may be markedly enhanced by NGF, a neurite-promoting factor.

Our results demonstrated that serum EPI levels of OAP rats were decreased in 2 weeks after birth. As the development of puberty, serum EPI increased gradually, and regained normal level. The expressions of PNMT protein in adrenal medulla showed the same tendency. However, ONP rats showed lower levels of serum EPI and the expressions of PNMT protein from birth to adulthood. The marker of AMCC is the synthesis and secretion of a large number of EPI, glucocorticoid induced expressions of synthetic enzyme PNMT that promoted the formation of EPI [26]–[28]. We presume that, although there existed impaired factors in asthma maternal uterus, adrenal medulla of offspring rats gradually were repaired during the development. We found that OVA-induced stress in asthma maternal rats during pregnancy enhanced significantly the serum levels of corticosterone in offspring rats from asthma and NGF maternal group from birth to adulthood. It is proved that the high glucocorticoids(GCs) concentrations in the adrenal medulla prevented the fiber outgrowth from medullary chromaffin cells in vivo [29] and contributed to the decrease of transformation adrenal cells into neurons [30], [31]. GCs could promote the expression of PNMT in adrenal medulla that catalyzes the conversion of norepinephrine to epinephrine [32]–[34].Thus we inferred that high corticosterone level may lead to the recovery of EPI level in offspring. However the increase of corticosterone level could not completely antagonize the alteration of NGF on the adrenal medulla that promoted chromaffin cells to differentiate into sympathetic nerve cells from birth to puberty, and exogenous GCs supplement may be essential.

Our results demonstrated that NGF antibody provided effects of repair on the adrenal medulla of offspring rats, which attributes mainly to the form of connective tissue that even divided adrenal medulla into island without influencing the alterations of its essential function.

In conclusion, our study partly suggested the fact that maternal asthma during pregnancy may promote AMCC to differentiate into sympathetic neurons in offspring rats, which inhibits the maturity of adrenal medulla, resulting in blocking EPI synthesis.

## Materials and Methods

### Experimental animals and preparation

All animals used in this study were 6 to 8-week-old male Sprague-Dawley rats (Experimental Animal Center of Central South University, Changsha, China) and all procedures performed on the animals were in compliance with the Chinese Council of Animal Care guidelines (approved by the Central South University Animal Care Committee).

Thirty-two pregnant rats were divided into four groups at random (*n* = 8 per group): control pregnant rats (CP), asthmatic pregnant rats (AP),NGF treated pregnant rats(NP),anti-NGF treated pregnant rats (ANP). The rats were treated as follows:[35], [36] on days 0 and 7, AP,NP and ANP rats were sensitized with an intraperitoneal injection of 100 mg of chicken OVA (Sigma, USA), 200 mg of aluminum hydroxide (Sigma, USA) and 6×10^9^ heat-killed Bordetella pertussis (Wuhan Institute of Biological Products, China) in 1 ml of sterile saline. The control rats were treated with a sterile saline intraperitoneal injection for sham sensitization. The sensitized rats were exposed to 30-minute of 1% OVA (wt/vol) aerosol every day from day 14 to day 21, while the control rats received filtered air only. NGF-7S (8 ng/kg, Sigma,USA, N0513) and its vehicle (PBS, 5 ml/kg) was injected intraperitoneally in NP rats 30 min before inhaling 1% OVA (wt/vol ) aerosol. Anti-NGF (1∶2,000 dilution, 4 ml/kg, Millipore, USA, 04-1119), and its vehicle (PBS, 4 ml/kg ) was injected intraperitoneally in ANP rats 30 min before inhaling 1% OVA (wt/vol) aerosol [13]. Male rats were select from every pregnant group offspring at random and were divided into four groups(*n* = 40 per group): offspring from control pregnant rats (OCP), offspring from asthmatic pregnant rats (OAP),offspring from NGF pregnant rats(ONP), offspring from anti-NGF pregnant rats (OANP).

### Measurement of bronchial responsiveness

In vivo airway responsiveness to histamine was measured 24 hours after the last OVA challenge using whole-body plethysmography (model PLY 3211; Buxco Electronics). Rats were treated for 2 minutes with aerosolized saline or increasing doses of histamine generated by an ultrasonic nebulizer, and airway resistance (RL) was measured. Histamine-induced bronchoconstriction was measured as the index of percent increase in airway resistance when compared to the peak of the reaction with baseline airway resistance.

### Bronchoalveolar lavage

After determination of bronchial responsiveness, the right main-stem bronchus was occluded with a clamp and the left lung was lavaged three times via a tracheal cannula with 3 ml volume of sterile saline. The bronchoalveolar lavage fluid (BALF) was recovered manually by gentle aspiration with a disposable syringe after each infusion; the recovery of BALF was >70%. The total cell numbers was estimated using a haemocytometer. The lavage fluid was centrifuged (4°C, 1000 r/min, 10 minutes), then the cytospin preparations were stained with May-Grunwald- Giemsa and differential cell counts were performed on a total of 200 cells.

### Transmission electron microscopy

Adrenal medullas were fixed with 2% glutaraldehyde in 0.1 M cacodylate buffer, pH 7.2. After 3 hours, specimens were post-fixed in buffered 1% OsO4 for 1 hour, dehydrated in ethanol, and embedded in Epon-Araldite. Ultrathin sections were stained with uranyl acetate and lead citrate and finally examined under a H-600 transmission electron microscope (Hitachi, Japan). The ultrastructure changes were assessed by pathologists who were blind to the treatment.

### Enzyme linked immunosorbent assay (ELISA)

Epinephrine(EPI), NGF, corticosterone levels in serum were quantified using the ELISA technique, utilizing commercially available antibodies, according to the protocol provided by the supplier (NGF, BPB Biomedical, BT555; Epinephrine, Serotec 0100-0009; corticosterone, Cayman, 500651-96). The reactions were read using an ELISA reader at 450 nm.

### Immunohistochemistry experiments

Rats were sacrificed by vertebral dislocation, and adrenal medulla was immediately removed and embedding in paraffin at 4°C overnight, tissues were sectioned (10 mm) and mounted on slides. Sections were then deparaffinized in toluene and rehydrated in ethanol with increasing concentrations of water. Quenching of endogenous peroxidase activity, incubation with antibodies and peroxidase staining were performed according to the manufacturer's instruction (ABC kit and NovaRED substrate kit, Zhongshang biologic company). Tissue sections were exposed to anti-PNMT antibody (Millipore USA, AB110, 1∶2500), and anti-NGF antibody (Millipore, USA, 04-1119, 1∶500) at 4°C overnight. Detection was achieved using AEC (3-amino-9-ethy-carbazole) kit as substrates, and nuclei were stained with Gill's hematoxylin. Negative controls were incubated in the absence of primary antibody.

### Statistics analysis

Values were expressed as mean ±SEM. At each age, the values for the control and prenatal treatment groups were compared using one-way ANOVA, followed by Fisher's protected least significant difference test. P value of<0.05 was considered significant.
